# Heterogeneous Renal Response to Therapy in Type 2 Diabetes: Albuminuria Categories

**DOI:** 10.3390/jcm15208181

**Published:** 2026-10-21

**Authors:** Rawan Jassim AlGhawi, Hesa Adel Albanki, Lama Mohammed Almahrous, Farah Ahmed Alabbasi, Mohammed Zurkiyeh, Anwar B Alotaibi, Ahmed M. Alkhunaizi

**Affiliations:** 1 Internal Medicine Department, Johns Hopkins Aramco Healthcare, Dhahran 34465, Saudi Arabia; 2 Research Department, Johns Hopkins Aramco Healthcare, Dhahran 34465, Saudi Arabia

**Keywords:** albuminuria, diabetes mellitus type 2, sodium-glucose transporter 2 inhibitors

## Abstract

**Background**: The comparative effectiveness of renin–angiotensin system (RAS) blockade and sodium-glucose cotransporter 2(SGLT2) inhibitors across different stages of albuminuria remain incompletely characterized despite demonstrating renoprotective association in randomized trials. This study evaluates the association between angiotensin-converting enzyme inhibitor (ACEI)/angiotensin receptor blocker (ARB), SGLT2 inhibitor, and their combination across baseline albuminuria strata. **Methods:** We conducted a retrospective cohort study at Johns Hopkins Aramco Healthcare including adults with type 2 diabetes with baseline and follow-up measurements of eGFR and urine albumin-to-creatinine ratio (ACR) between January 2020 and December 2024. Patients were categorized into the following four groups: neither therapy, ACEI/ARB alone, SGLT2 inhibitor alone, and combination therapy. Analyses were stratified by baseline albuminuria category. **Results:** Among 10,252 participants, treatment groups comprised neither ACEI/ARB nor SGLT2 inhibitor therapy (*n* = 2142), ACEI/ARB monotherapy (*n* = 2332), SGLT2 inhibitor monotherapy (*n* = 1988), and combination therapy (*n* = 3790). In the entropy-balanced adjusted analysis, none of the treatment groups were significantly associated with the primary outcome of ≥30% eGFR decline compared with neither therapy. For the secondary outcome of ≥10% eGFR decline, higher adjusted odds were observed with ACEI/ARB monotherapy (OR, 1.48; 95% CI, 1.24–1.75) and combination therapy (OR, 1.62; 95% CI, 1.39–1.89), whereas SGLT2 inhibitor monotherapy was not significantly associated with the outcome. In the baseline-adjusted log-ACR analysis, higher adjusted geometric mean follow-up ACR was observed with ACEI/ARB monotherapy (GMR, 1.079; 95% CI, 1.007–1.155) and combination therapy (GMR, 1.203; 95% CI, 1.128–1.284); the association for SGLT2 inhibitor monotherapy was not statistically significant. In a binary threshold analysis, no treatment groups demonstrated a statistically significant association with achieving a ≥50% ACR decline compared to neither therapy. A significant treatment-by-baseline-ACR interaction was observed for ≥30% eGFR decline (*p* = 0.029). **Conclusions:** Overall, treatment group was not significantly associated with the primary outcome of ≥30% eGFR decline or a categorical ≥50% decline in ACR in the adjusted analysis. However, the significant interaction with baseline ACR indicates that the association between treatment group and eGFR decline varied across albuminuria categories. Variations in continuous eGFR change and baseline-adjusted continuous follow-up ACR differed across treatment groups, showing slight increases in geometric mean follow-up ACR for groups exposed to ACEI/ARBs.

## 1. Introduction

Despite advances in diabetes care, diabetic kidney disease (DKD) remains a leading cause of chronic kidney disease worldwide and a major determinant of cardiovascular outcomes [1,2]. Its progression is often clinically silent, characterized by a prolonged asymptomatic phase followed by a gradual yet irreversible decline in kidney function [1]. The prognostic interplay between declining estimated glomerular filtration rate (eGFR) and increasing albuminuria is well-established, with both markers independently and synergistically predicting kidney failure and mortality [3,4]. Moreover, modest reductions in albuminuria or attenuation in eGFR decline have been associated with improved long-term renal and cardiovascular outcomes [3,4].

Inhibition of the renin–angiotensin system (RAS) with angiotensin-converting enzyme inhibitors (ACEIs) or angiotensin receptor blockers (ARBs) constitutes the cornerstone of renoprotective therapy in albuminuric diabetes [5,6,7,8]. Landmark randomized trials demonstrated that RAS blockades reduce proteinuria and delay progression to advanced kidney outcomes beyond their antihypertensive association [5,6]. However, despite these benefits, a substantial residual risk persists, and progressive decline in kidney function remains common in patients with established DKD [1].

The therapeutic paradigm has evolved with the emergence of sodium-glucose cotransporter 2(SGLT2) inhibitors, which confer renoprotective association beyond glycemic control through modulation of intraglomerular hemodynamics, reduction in hyperfiltration, and attenuation of inflammatory and fibrotic pathways [9,10,11]. Large randomized trials and meta-analyses, including Canagliflozin and Renal Events in Diabetes with Established Nephropathy Clinical Evaluation (CREDENCE), Dapagliflozin and Prevention of Adverse Outcomes in Chronic Kidney Disease (DAPA-CKD), the Study of Heart and Kidney Protection with Empagliflozin (EMPA-KIDNEY), and pooled analyses of cardiovascular outcome trials, have consistently demonstrated that SGLT2 inhibitors reduce kidney disease progression across broad populations with type 2 diabetes and CKD [10,11,12,13]. Contemporary Kidney Disease: Improving Global Outcomes (KDIGO) and American Diabetes Association (ADA)-KDIGO guidance now position SGLT2 inhibitors, together with RAS blockade when indicated, as foundational therapy in diabetic CKD [1,14,15].

Recently, nonsteroidal mineralocorticoid receptor antagonists, particularly finerenone, have further expanded the therapeutic landscape by demonstrating additional reductions in albuminuria and kidney disease progression when added to standard of care, including RAS inhibition [16,17]. These advances reflect a shift toward multidrug strategies targeting complementary pathophysiologic pathways in DKD.

However, while randomized trials establish efficacy under controlled conditions, translating this evidence into routine clinical practice remains complex. Real-world populations are heterogeneous, baseline kidney function and albuminuria burden vary substantially, and treatment allocation is inherently nonrandom [18]. Although evidence supports the kidney-protective association of SGLT2 inhibitors, there is a gap in the existing literature regarding the longitudinal effectiveness of combined ACEI/ARB and SGLT2 inhibitor therapy compared with monotherapy or no exposure to either agent in routine clinical practice [18].

We also examined whether associations between groups and kidney outcomes differed across baseline albuminuria categories. Understanding whether dual therapy confers incremental renoprotection, and whether this benefit is reflected in the trajectories of the albumin-to-creatinine ratio (ACR) and eGFR decline, has important implications for therapeutic prioritization and health system planning. This longitudinal cohort study aims to evaluate renal outcomes among patients with type 2 diabetes stratified by exposure to ACEI/ARB therapy, SGLT2 inhibitor therapy, both agents, or neither through assessing changes in ACR and eGFR over time, in addition to determining the real-world comparative effectiveness of combination therapy in modifying renal trajectories relative to monotherapy or no exposure.

## 2. Materials and Methods

### 2.1. Study Design and Setting

This retrospective cohort study was conducted at Johns Hopkins Aramco Healthcare (JHAH) to evaluate longitudinal renal outcomes among adults with type 2 diabetes mellitus. Data were extracted from the EPIC electronic health record system Corporation (Verona, WI, USA) and covered the period from January 2020 to December 2024. The study was approved by the JHAH Institutional Review Board (approval code: 25-01-153; date of approval: 7 April 2025). Due to the retrospective nature of the study and the use of de-identified data, informed consent was waived.

### 2.2. Study Population

Adults aged ≥18 years with a documented diagnosis of type 2 diabetes mellitus and available baseline and follow-up renal assessments were included. Baseline was defined as the earliest qualifying laboratory measurement recorded in calendar year 2020. Follow-up was defined as the most recent available renal assessment through December 2024. When 2024 data were unavailable, the last recorded value between 2021 and 2024 was used. Inclusion criteria required measurement of serum creatinine and urine albumin-to-creatinine ratio (ACR, mg albumin/g creatinine) at baseline (calendar year 2020) and at least one follow-up assessment between 2021 and the end of 2024. Patients with type 1 diabetes mellitus, gestational diabetes, steroid-induced diabetes, and those younger than 18 years were excluded.

A total of 10,252 patients were included and categorized into the following four treatment groups: neither therapy (*n* = 2142), ACEI or ARB therapy alone (*n* = 2332), SGLT2 inhibitor therapy alone (*n* = 1988), and combined ACEI/ARB and SGLT2 inhibitor therapy (*n* = 3790). Patients were further stratified by baseline albuminuria category as follows: normal (<30 mg/g; *n* = 7316), microalbuminuria (30–300 mg/g; *n* = 2300), and macroalbuminuria (>300 mg/g; *n* = 636). Patients who died during follow-up contributed data up to the time of death. Missing follow-up laboratory values were handled using available-case analysis.

### 2.3. Exposure Definition

Medication exposure was defined by an active prescription documented in 2020. Disease progression was defined as a ≥30% decline in eGFR from baseline, consistent with established surrogate endpoints used in CKD research. Participants were assigned to one of the following four groups: ACEI or ARB therapy, SGLT2 inhibitor therapy, combined ACEI/ARB and SGLT2 inhibitor therapy, and neither therapy.

### 2.4. Assessment of Kidney Function

Kidney function was assessed using both estimated creatinine clearance (CrCl) and estimated glomerular filtration rate (eGFR) calculated from serum creatinine measurements. CrCl was calculated using the Cockcroft–Gault equation:

$$\mathrm{CrCl}\;(\mathrm{mL}/\min)=\frac{\left(140-\mathrm{age}\right)\times \mathrm{weight}(\mathrm{kg})}{72\times \mathrm{serum creatinine}\;(\mathrm{mg}/\mathrm{dL})}\times [0.85\;\mathrm{if female}]$$

eGFR was determined using the Chronic Kidney Disease Epidemiology Collaboration (CKD-EPI) equation. Serum creatinine concentrations were measured using a clinical laboratory assay standardized to isotope dilution mass spectrometry (IDMS) traceable standards, which is required for accurate estimation with the CKD-EPI formula.

### 2.5. Outcomes

#### 2.5.1. Primary Outcomes

The primary outcome was a ≥30% decline in estimated glomerular filtration rate (eGFR) from baseline to follow-up. The outcome was coded as a binary variable, with participants meeting the threshold classified as having experienced the outcome and those not meeting the threshold classified as not having experienced it.

#### 2.5.2. Secondary Outcomes

Secondary outcomes included a ≥10% decline in eGFR, continuous change in eGFR, and baseline-adjusted follow-up urine albumin-to-creatinine ratio (ACR). The ≥10% eGFR decline outcome was defined using the same baseline-to-follow-up comparison as the primary outcome, with a threshold of 10%.

Continuous change in eGFR was calculated as follow-up eGFR minus baseline eGFR and analyzed as a continuous outcome. ACR was analyzed using a baseline-adjusted model of log-transformed follow-up ACR because the distribution of ACR was highly right-skewed. Participants with non-positive baseline or follow-up ACR values were excluded from the log-transformed analysis because logarithms are not defined for non-positive values. The model included baseline log-transformed ACR, age, sex, body mass index (BMI), baseline HbA1c, and baseline eGFR as covariates. Treatment-group coefficients from this model were exponentiated and reported as adjusted geometric-mean ratios (GMRs) with 95% confidence intervals (CIs). A GMR greater than 1 indicates a higher adjusted geometric mean follow-up ACR relative to the reference group, conditional on the model covariates.

The treatment groups were ACEI/ARB monotherapy, SGLT2 inhibitor monotherapy, combination therapy, and neither therapy. The neither-therapy group served as the reference category for all treatment comparisons. Analyses used the available observations meeting the eligibility and completeness requirements for each outcome model.

Additionally, a binary secondary endpoint was defined as achieving a ≥50% reduction in ACR from baseline to follow-up.

### 2.6. Covariates

Analyses adjusted for clinically relevant baseline covariates included age, sex, body mass index (BMI), hemoglobin A1c (HbA1c), baseline eGFR, and baseline ACR.

### 2.7. Statistical Analysis

Baseline characteristics were summarized by treatment group and baseline ACR category. Entropy balancing was used to derive weights intended to improve balance in measured baseline covariates across treatment groups. The covariate balance achieved by the weighting procedure was assessed before outcome modeling.

Associations between treatment group and the primary binary outcome of ≥30% eGFR decline were estimated using weighted regression, with neither ACEI/ARB nor SGLT2 inhibitor therapy as the reference group. Adjusted odds ratios (ORs) and 95% CIs were reported. The same modeling approach was applied to the secondary binary outcome of ≥10% eGFR decline. Fractional-weight regression models used a quasibinomial family where appropriate.

Continuous change in eGFR was analyzed using a weighted regression model, with treatment group as the main explanatory variable and adjustment for the prespecified baseline covariates. For ACR, a weighted linear regression model was fitted with log-transformed follow-up ACR as the dependent variable. The model adjusted for baseline log-transformed ACR, age, sex, BMI, baseline HbA1c, and baseline eGFR. Exponentiated treatment coefficients were reported as adjusted GMRs with 95% CIs.

Effect modification by baseline ACR category was assessed by including interaction terms between treatment group and baseline ACR category in the model for the primary outcome. A global test was used to assess the overall treatment-by-ACR interaction. Individual interaction terms were reported with their ORs, 95% CIs, and *p*-values. These terms were interpreted as measures of effect heterogeneity relative to the specified reference categories, rather than as stratum-specific treatment effects.

All analyses were conducted in R (version 4.5). Statistical tests were two-sided, and *p* < 0.05 was considered statistically significant.

## 3. Results

### 3.1. Baseline Characteristics

A total of 10,252 participants were included in the analysis. Of these, 2142 (20.9%) received neither ACEI/ARB nor SGLT2 inhibitor therapy, 2332 (22.7%) received ACEI/ARB therapy alone, 1988 (19.4%) received SGLT2 inhibitor therapy alone, and 3790 (37.0%) received both therapies (Table 1).

Baseline characteristics differed across treatment groups. Participants receiving ACEI/ARB therapy alone or combination therapy were older and had lower baseline eGFR than those receiving neither therapy, whereas participants receiving SGLT2 inhibitor therapy alone had higher baseline HbA1c. Baseline ACR was also higher among participants receiving combination therapy. The proportion of participants with microalbuminuria or macroalbuminuria was greater among those receiving ACEI/ARB therapy alone or combination therapy than among those receiving neither therapy nor SGLT2 inhibitor therapy alone.

### 3.2. Primary Outcome: ≥30% Decline in eGFR

In the entropy-balanced adjusted analysis, treatment group was not significantly associated with the primary outcome of a ≥30% decline in eGFR (Table 2). Compared with participants receiving neither ACEI/ARB nor SGLT2 inhibitor therapy, the adjusted odds ratio (OR) was 1.05 (95% CI, 0.71–1.55; *p* = 0.792) for ACEI/ARB therapy alone, 0.76 (95% CI, 0.44–1.31; *p* = 0.319) for SGLT2 inhibitor therapy alone, and 1.36 (95% CI, 0.98–1.87; *p* = 0.063) for combination therapy.

### 3.3. Continuous Change in eGFR and Follow-Up ACR

#### 3.3.1. Change in eGFR

In the adjusted analysis of continuous eGFR change, ACEI/ARB therapy alone was associated with a −1.28 mL/min/1.73 m^2^ difference compared with neither therapy (SE, 0.49; *p* = 0.008), whereas SGLT2 inhibitor therapy alone was not significantly different from the reference group (β = 0.42; SE, 0.55; and *p* = 0.445). Combination therapy was associated with a −1.89 mL/min/1.73 m^2^ difference compared with neither therapy (SE, 0.44; *p* < 0.001) (Table 3).

#### 3.3.2. Change in ACR

For ACR percentage change, ACEI/ARB therapy alone and SGLT2 inhibitor therapy alone were not significantly different from the reference group. Combination therapy showed a positive adjusted percentage change in ACR (β = 72.35%; SE, 33.79; and *p* = 0.032). Given the highly skewed distribution of ACR and the potential influence of extreme values, this finding was interpreted cautiously and considered alongside the log-transformed ACR and descriptive analyses (Table 3).

#### 3.3.3. ≥10% Decline in eGFR

For the secondary threshold of a ≥10% decline in eGFR, ACEI/ARB therapy alone and combination therapy were associated with higher adjusted odds of the outcome compared with neither therapy (Table 4). The adjusted OR was 1.48 (95% CI, 1.24–1.75; *p* < 0.001) for ACEI/ARB therapy alone and 1.62 (95% CI, 1.39–1.89; *p* < 0.001) for combination therapy. SGLT2 inhibitor therapy alone was not significantly different from the reference group (OR, 0.97; 95% CI, 0.78–1.21; and *p* = 0.774).

#### 3.3.4. ≥50% Decline in ACR

After adjusting for baseline clinical characteristics using entropy balancing weights, the corrected logistic regression model demonstrated no statistically significant differences across any treatment groups in achieving a robust ≥50% decline in ACR. Compared to the untreated reference cohort (Neither), the odds of achieving a ≥50% ACR reduction were comparable for patients receiving ACEI/ARB monotherapy (Adjusted OR: 1.02; 95% CI: 0.84–1.24; and *p* = 0.857), SGLT2 inhibitor monotherapy (Adjusted OR: 1.03; 95% CI: 0.81–1.32; and *p* = 0.798), and combination therapy (Adjusted OR: 0.95; 95% CI: 0.80–1.13; and *p* = 0.549) (Table 4).

The forest plot summarizes adjusted associations between treatment groups and renal outcomes, using the neither-therapy group as the reference. For the primary outcome of a ≥30% eGFR decline, no treatment groups show a statistically significant association as all confidence intervals span across the null line at 1.0. In contrast, the secondary acute threshold of a ≥10% eGFR decline reveals significantly higher adjusted odds for both the ACEI/ARB monotherapy and combination therapy groups. Crucially, the corrected model for a ≥50% ACR decline shows completely neutral, non-significant associations across all treatment arms, successfully resolving the previous clinical paradox by demonstrating that all error bars safely cross 1.0 (Figure 1).

### 3.4. Effect Modification by Baseline ACR

A statistically significant interaction was observed between treatment group and baseline ACR category for the primary outcome of a ≥30% decline in eGFR (global treatment-by-ACR interaction *p* = 0.029; Table 5). This indicates that the association between treatment group and the primary renal outcome varied according to baseline albuminuria status.

Using neither ACEI/ARB nor SGLT2 inhibitor therapy and normal baseline ACR (<30 mg/g) as the reference categories, the interaction terms for ACEI/ARB therapy with microalbuminuria and combination therapy with microalbuminuria were statistically significant (OR, 0.351; 95% CI, 0.137–0.903; and *p* = 0.030 and OR, 0.209; 95% CI, 0.095–0.459; and *p* < 0.001, respectively). The interaction between SGLT2 inhibitor therapy and microalbuminuria was not statistically significant (OR, 0.389; 95% CI, 0.132–1.140; and *p* = 0.086).

For macroalbuminuria, the interaction terms were not statistically significant for ACEI/ARB therapy (OR, 0.407; 95% CI, 0.139–1.190; and *p* = 0.100), whereas statistically significant interaction terms were observed for SGLT2 inhibitor therapy (OR, 0.178; 95% CI, 0.040–0.796; and *p* = 0.024) and combination therapy (OR, 0.202; 95% CI, 0.079–0.517; and *p* < 0.001).

These findings indicate heterogeneity in the association between the treatment group and the primary renal outcome across baseline ACR categories. The interaction estimates represent treatment-by-ACR interaction terms and should therefore not be interpreted as stratum-specific treatment effects. Detailed stratum-specific analyses are provided in the Supplementary Materials.

While the interaction terms for the macroalbuminuria subgroup achieved statistical significance, these stratum-specific findings are based on a highly restricted sample size (*n* = 34) and small event counts, requiring substantial caution in interpretation.

## 4. Discussion

In this large real-world cohort of patients with type 2 diabetes, treatment group was not significantly associated with the primary outcome of a ≥30% decline in eGFR in the overall entropy-balanced analysis. However, a significant treatment-by-baseline ACR interaction was observed, indicating that the association between renoprotective therapy and kidney outcomes varied according to baseline albuminuria status. Significant interaction terms were observed for ACEI/ARB and combination therapy in patients with microalbuminuria and for SGLT2 inhibitor and combination therapy in patients with macroalbuminuria. These findings support heterogeneity in renal treatment associations across albuminuria categories, while the interaction estimates themselves should not be interpreted as direct stratum-specific treatment effects. In contrast, treatment associations were less consistent in microalbuminuria and appeared paradoxical in patients with normal baseline ACR. These findings suggest that baseline albuminuria may modify the association between renoprotective therapy and renal outcomes, highlighting the importance of considering disease stage when evaluating treatment response.

The observed heterogeneity in treatment associations across albuminuria categories is biologically plausible. Albuminuria is not only a marker of kidney injury but also a prognostic indicator of future kidney and cardiovascular outcomes [3,4,19]. Patients with macroalbuminuria generally have more advanced glomerular injury, intraglomerular hypertension, endothelial dysfunction, and activation of inflammatory and fibrotic pathways [19,20]. These mechanisms are targeted by RAS blockade, SGLT2 inhibitors, and newer agents such as finernone [5,6,10,11,16,17]. Prior randomized trials have demonstrated kidney-protective effects of these therapies across populations at elevated kidney risks [10,11,13,16,17]. These data provide biological context for the heterogeneity observed in our analysis, although the present observational findings do not establish differential causal treatment effects across albuminuria strata.

The significant interaction involving SGLT2 inhibitor therapy and macroalbuminuria is biologically plausible in the context of the established kidney-protective mechanisms of this drug use. SGLT2 inhibitors reduce proximal tubular sodium-glucose reabsorption, restore tubuloglomerular feedback, lower intraglomerular pressure, and attenuate hyperfiltration [9,21]. In CREDENCE, DAPA-CKD, and EMPA-KIDNEY, SGLT2 inhibitors significantly reduced kidney disease progression across broad CKD populations, including patients with diabetes and albuminuria [10,11,13]. These trial findings provide biological and clinical context for the heterogeneity observed in our analysis, although the observational design and interaction-based estimates in the present study do not establish stratum-specific causal treatment effects.

The magnitude of eGFR decline observed in this cohort should be interpreted in the context of the expected natural history of DKD. In healthy adults, eGFR may decline by approximately 0.8–1 mL/min/1.73 m^2^ per year with aging, whereas in type 2 diabetes, reported median eGFR slopes are often faster, approximately −1.5 to −4.0 mL/min/1.73 m^2^ per year [22]. Additionally, KDIGO defines a decline of ≥5 mL/min/1.73 m^2^ per year as rapid progression [1,22]. Over 5 years, this corresponds to a clinically relevant expected decline, especially in patients with albuminuria. In the present cohort, treatment groups differed in continuous eGFR change and in the odds of a ≥10% decline in eGFR, whereas no significant overall treatment association was observed for the primary ≥30% decline outcome. These apparently differing findings across renal endpoints emphasize the complexity of interpreting kidney-function trajectories in observational data and the potential influence of baseline disease severity and residual confounding. Furthermore, an early hemodynamic decline in eGFR following ACEI/ARB or SGLT2 inhibitor initiation does not necessarily indicate progressive kidney injury; however, the baseline-to-follow-up measurements used in this study could not distinguish this initial response from sustained deterioration in kidney function.

The microalbuminuria group represents an intermediate phenotype between normal albumin excretion and established macroalbuminuric DKD. Contrary to macroalbuminuria, which often reflects more advanced and sustained glomerular injury, microalbuminuria may represent earlier, more variable, and potentially reversible kidney injury, which explains the less pronounced treatment association in this group compared to the macroalbuminuria group [19,23]. Within the interaction analysis, significant treatment-by-microalbuminuria terms were observed for ACEI/ARB therapy and combination therapy, whereas the interaction involving SGLT2 inhibitor therapy was not statistically significant. These findings suggest that baseline microalbuminuria modified the association between some treatment categories and the primary renal outcome, although the interaction estimates should not be interpreted as direct treatment effects within the microalbuminuria stratum [1,14,15].

On the other hand, the apparent higher risk of eGFR decline among treated patients with normal baseline ACR should not be interpreted as evidence of harm. Patients with normal ACR who were prescribed renoprotective medications likely differed from untreated patients in ways not fully captured by available covariates. They may have had elevated cardiovascular risk, prolonged durations of diabetes, hypertension, history of vascular disease, or clinician-perceived risk that prompted therapeutic intervention despite normal albuminuria. Although statistical adjustment reduces measured baseline imbalance, it cannot eliminate residual confounding from unmeasured factors [24,25]. Therefore, confounding by indication is a plausible explanation for the apparent higher risk observed among treated patients with normal ACR, although residual confounding cannot be excluded.

Albuminuria outcomes require cautious interpretation. ACR is highly biologically variable, right-skewed, and sensitive to outliers, hydration status, glycemic control, blood pressure, intercurrent illness, and timing of urine collection [23,26]. In the adjusted analysis, ACEI/ARB and SGLT2 inhibitor monotherapy were not significantly associated with percentage change in ACR, whereas combination therapy showed a significant positive adjusted percentage change. However, given the marked skewness of ACR and potential influence of extreme values, this finding should be interpreted cautiously. In the secondary threshold analysis of ≥50% ACR decline, no treatment groups demonstrated a statistically significant association with achieving this endpoint compared to the untreated reference group, highlighting that threshold-based responses were uniform across cohorts. Together, these findings illustrate the sensitivity of albuminuria outcomes to the analytical approach and argue against drawing strong conclusions from any single ACR measure [27].

The finding that a subset of patients with microalbuminuria and macroalbuminuria were not receiving ACEI/ARB or SGLT2 inhibitor therapy is clinically important. Current KDIGO and ADA-KDIGO recommendations support RAS blockade for patients with diabetes, hypertension, and albuminuria and recommend SGLT2 inhibitors for most patients with type 2 diabetes and CKD who meet eGFR criteria [1,14,15]. Therefore, absence of therapy among eligible albuminuria patients may represent underutilization of guideline-directed kidney-protective therapy. This observation identifies a potential quality-improvement opportunity at the health-system level, including earlier recognition of albuminuria, clinician alerts, medication optimization pathways, and follow-up systems to ensure appropriate initiation and continuation of therapy [28,29].

The predominance of male patients in this cohort reflects the demographic structure of the JHAH population, which mainly consists of Saudi Aramco employees and beneficiaries; a workforce historically composed predominantly of males. Therefore, the higher male percentage is attributable to the occupational composition rather than disease-specific sex differences. Although sex distribution did not differ significantly across albuminuria strata in this cohort, future studies with broader community-based sampling may better assess whether these findings are generalizable to women and non-industrial populations.

### Strengths and Limitations

This study has several strengths, including its large sample size, longitudinal follow-up, real-world setting, stratification by baseline albuminuria, and use of advanced statistical adjustment to reduce measured confounding. The real-world design reflects routine clinical practice rather than the controlled conditions of placebo-based randomized trials. In everyday care, patients may miss doses, discontinue medications, switch therapies, or receive treatment based on clinician judgment and comorbid risk. Therefore, these findings provide practical insight into how renoprotective therapies perform outside trial settings. The use of stratified analyses also allowed treatment association to be interpreted within clinically meaningful albuminuria categories rather than assuming a uniform association across all patients with diabetes. However, several limitations should be acknowledged. The retrospective observational design limits causal inference and introduces the possibility of residual confounding despite statistical adjustment [24,25]. Medication exposure was based on prescription data; therefore, actual adherence, dose intensity, treatment interruption, missed doses, and discontinuation could not be reliably confirmed. Additionally, non-prescription of ACEI/ARB or SGLT2 inhibitor therapy among patients with albuminuria may reflect clinical contraindications, intolerance, patient preference, or prescribing gaps that could not be fully captured in the available data. ACR variability and regression to the mean may have influenced descriptive albuminuria outcomes, particularly the proportion achieving ≥50% ACR reduction, which was reported descriptively rather than being modeled due to the extreme skewness and outlier influence of ACR values [23,26,27]. Moreover, the macroalbuminuria subgroup was smaller than the normal and microalbuminuria groups, limiting precision for some comparisons. In addition, the predominantly male, occupationally linked population may limit generalizability to other healthcare settings. Furthermore, significant treatment-by-ACR interaction terms indicate heterogeneity across albuminuria categories but should not be interpreted as direct stratum-specific treatment effects; therefore, subgroup findings should be considered exploratory and interpreted cautiously.

## 5. Conclusions

In this real-world cohort of patients with type 2 diabetes, treatment group was not significantly associated with the primary outcome of ≥30% eGFR decline in the overall adjusted analysis. However, the significant treatment-by-ACR interaction indicates that renal treatment associations varied according to baseline albuminuria status. Significant interaction terms involving ACEI/ARB and combination therapy in microalbuminuria, and SGLT2 inhibitor and combination therapy in macroalbuminuria, support potential heterogeneity across stages of albuminuria, though these latter subgroup findings should be interpreted with caution due to limited sample sizes and event counts, and these estimates should not be interpreted as direct stratum-specific treatment effects. These findings highlight baseline albuminuria as an important factor when evaluating renal outcomes in routine clinical practice and support further prospective studies to clarify treatment effects across the spectrum of diabetic kidney disease.

## Figures and Tables

**Figure 1:**
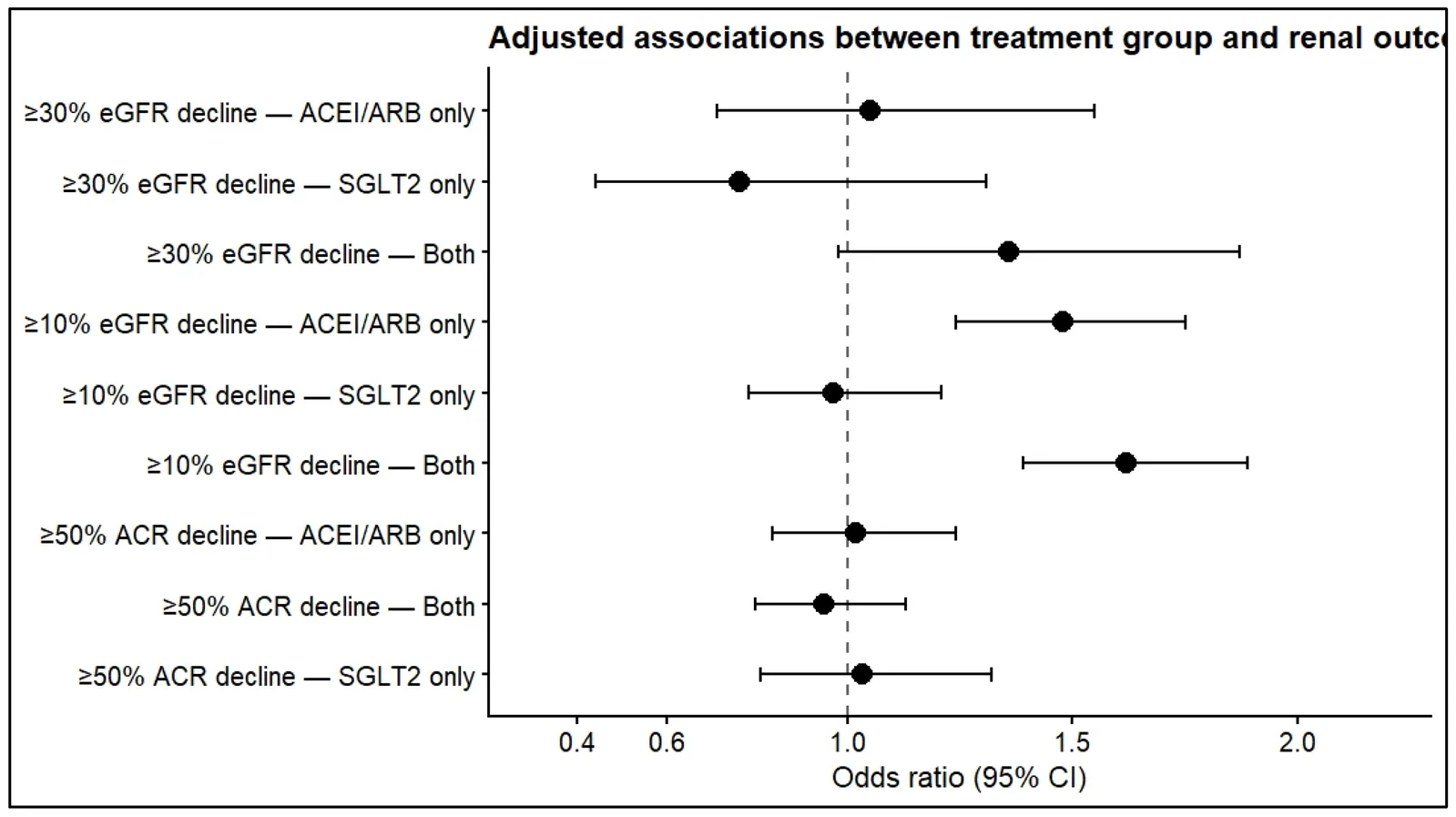
Adjusted associations between treatment group and renal outcomes in the overall study population. Odds ratios and 95% confidence intervals are shown relative to neither therapy.

**Table 1:** Baseline characteristics of the study population according to treatment group.

Characteristic	Neither (*n* = 2142)	ACEI/ARB Only (*n* = 2332)	SGLT2 Only (*n* = 1988)	Both (*n* = 3790)	*p* Value
**Age, years, mean ± SD**	60.4 ± 12.9	66.8 ± 10.3	59.9 ± 10.6	65.2 ± 9.7	<0.001
**BMI, kg/m^2^, mean ± SD**	29.8 ± 5.9	30.8 ± 6.0	29.6 ± 5.5	30.5 ± 5.7	<0.001
**Male sex, n (%)**	1104 (51.5)	1210 (51.9)	1051 (52.9)	2126 (56.1)	<0.001
**Baseline HbA1c, %, mean ± SD**	7.04 ± 1.55	7.07 ± 1.48	8.10 ± 1.77	8.13 ± 1.70	<0.001
**Baseline eGFR, mL/min/1.73 m^2^, mean ± SD**	96.5 ± 19.6	88.6 ± 19.1	99.0 ± 16.1	89.2 ± 20.2	<0.001
**Baseline ACR, mg/g, median [IQR]**	8.2 [5.1–16.7]	13.9 [6.8–41.0]	9.0 [5.6–17.3]	23.2 [9.1–83.4]	<0.001
**Coronary artery disease, n (%)**	192 (9.0)	419 (18.0)	237 (11.9)	918 (24.2)	<0.001
**Neuropathy, n (%)**	39 (1.8)	80 (3.4)	57 (2.9)	163 (4.3)	<0.001
**Baseline ACR category, n (%)**					
**Normal (<30 mg/g)**	1847 (86.2)	1625 (69.7)	1717 (86.4)	2127 (56.1)	<0.001
**Micro (30–300 mg/g)**	241 (11.3)	573 (24.6)	237 (11.9)	1249 (33.0)	
**Macro (>300 mg/g)**	54 (2.5)	134 (5.7)	34 (1.7)	414 (10.9)	

ACR, albumin-to-creatinine ratio; ACEI, angiotensin-converting enzyme inhibitor; ARB, angiotensin II receptor blocker; BMI, body mass index; eGFR, estimated glomerular filtration rate; HbA1c, glycated hemoglobin; SGLT2, sodium-glucose cotransporter 2.

**Table 2:** Adjusted association between treatment group and ≥30% decline in eGFR.

Treatment Group	Adjusted OR	95% CI	*p* Value
**ACEI/ARB only**	1.05	0.71–1.55	0.792
**SGLT2 only**	0.76	0.44–1.31	0.319
**Both**	1.36	0.98–1.87	0.063

ACEI, angiotensin-converting enzyme inhibitor; ARB, angiotensin II receptor blocker; OR, odds ratio; SGLT2, sodium-glucose cotransporter 2.

**Table 3:** Adjusted change in eGFR and ACR according to treatment group.

**(A) Change in eGFR**			
**Treatment group**	**Adjusted β**	**SE**	***p* value**
**ACEI/ARB only**	−1.28	0.49	0.008
**SGLT2 only**	0.42	0.55	0.445
**Both**	−1.89	0.44	<0.001
**(B) Change in ACR**			
**Treatment group**	**Adjusted β (% change)**	**SE**	***p* value**
**ACEI/ARB only**	7.02	15.39	0.648
**SGLT2 only**	−5.19	15.87	0.744
**Both**	72.35	33.79	0.032

ACR, albumin-to-creatinine ratio; ACEI, angiotensin-converting enzyme inhibitor; ARB, angiotensin II receptor blocker; eGFR, estimated glomerular filtration rate; SGLT2, sodium-glucose cotransporter 2; SE, standard error.

**Table 4:** Adjusted associations with ≥10% eGFR decline and ≥50% ACR decline.

Outcome/Treatment Group	Adjusted OR	95% CI	*p* Value
**≥10% eGFR decline**			
**ACEI/ARB only**	1.48	1.24–1.75	<0.001
**SGLT2 only**	0.97	0.78–1.21	0.774
**Both**	1.62	1.39–1.89	<0.001
**≥50% ACR decline**			
**ACEI/ARB only**	1.02	0.84–1.24	0.857
**SGLT2 only**	1.03	0.81–1.32	0.798
**Both**	0.95	0.80–1.13	0.549

ACEI, angiotensin-converting enzyme inhibitor; ARB, angiotensin II receptor blocker; eGFR, estimated glomerular filtration rate; OR, odds ratio; SGLT2, sodium-glucose cotransporter 2.

**Table 5:** Interaction between treatment group and baseline ACR category for ≥30% eGFR decline.

Interaction Term	OR	95% CI	*p* Value
**ACEI/ARB × Microalbuminuria**	0.351	0.137–0.903	0.030
**SGLT2 × Microalbuminuria**	0.389	0.132–1.140	0.086
**Both × Microalbuminuria**	0.209	0.095–0.459	<0.001
**ACEI/ARB × Macroalbuminuria**	0.407	0.139–1.190	0.100
**SGLT2 × Macroalbuminuria**	0.178	0.040–0.796	0.024
**Both × Macroalbuminuria**	0.202	0.079–0.517	<0.001

Global treatment × ACR interaction: Rao–Scott F test, *p* = 0.0287. Reference categories: Treatment: Neither ACR: Normal (<30 mg/g); ACEI, angiotensin-converting enzyme inhibitor; ARB, angiotensin II receptor blocker; eGFR, estimated glomerular filtration rate; OR, odds ratio; SGLT2, sodium-glucose cotransporter 2.

## Data Availability

The data that support the findings of this study are available from the corresponding author upon reasonable request, subject to institutional data sharing agreements and applicable data privacy regulations.

## Supplementary Materials

The following supporting information can be downloaded at https://www.mdpi.com/article/10.3390/jcm15208181/s1, Table S1: baseline characteristics by ACR; Table S2: unadjusted eGFR outcomes; Table S3: ACR outcomes; Table S4: pairwise treatment comparisons; Table S5: Firth sensitivity analysis; Table S6: weighted descriptive outcomes; Table S7: overall Firth sensitivity analysis; Table S8: treatment vs. ACR interaction; Table S9: entropy-balancing diagnostics.

## Abbreviations

The following abbreviations are used in this manuscript:

DKD	diabetic kidney disease
eGFR	estimated glomerular filtration rate
RAS	renin–angiotensin system
ACEIs	angiotensin-converting enzyme inhibitors
ARBs	angiotensin receptor blockers
SGLT2	sodium-glucose cotransporter 2
ACR	albumin-to-creatinine ratio
