# Supplementary material for: Heterogeneous Renal Response to Therapy in Type 2 Diabetes: Albuminuria Categories
Source: J Clin Med. 2026 Oct 21;15(20):8181. doi: 10.3390/jcm15208181 (PMC13706043; doi:10.3390/jcm15208181)
Supplement: Supplementary file 1 [file jcm-15-08181-s001.zip › jcm-4515955-supplementary.pdf]

Baseline ACR categories differed substantially in baseline renal and clinical characteristics. Higher ACR categories were accompanied by higher HbA1c, lower baseline eGFR, and higher prevalence of CAD and neuropathy. Treatment distribution also differed across ACR categories, with combination therapy more frequent among participants with microalbuminuria and macroalbuminuria. These differences support the use of covariate adjustment and treatment-group weighting in the primary analyses **(Table S1).**

Supplementary Table S1. Baseline characteristics according to baseline ACR category

Characteristic	Normal ACR (<30 mg/g)	Microalbuminuria (30–300 mg/g)	Macroalbuminuria (>300 mg/g)
**N**	7,316	2,300	636
**Age, years**	62.7	65.2	67.9
**Male sex, n (%)**	3,922 (53.6)	1,235 (53.7)	336 (52.8)
**BMI, kg/m²**	30.1	30.6	30.9
**HbA1c, %**	7.5	8.0	8.4
**Baseline eGFR, mL/min/1.73 m²**	95.0	89.9	73.1
**Baseline ACR, mg/g, median**	8.5	65.7	702.2
**CAD, n (%)**	1,068 (14.6)	499 (21.7)	201 (31.6)
**Neuropathy, n (%)**	197 (2.7)	97 (4.2)	48 (7.5)
**Neither therapy, n (%)**	1,847 (25.2)	241 (10.5)	54 (8.5)
**ACEI/ARB only, n (%)**	1,625 (22.2)	573 (24.9)	134 (21.1)
**SGLT2 only, n (%)**	1,717 (23.5)	237 (10.3)	34 (5.3)
**Both therapies, n (%)**	2,127 (29.1)	1,249 (54.3)	414 (65.1)

ACR, albumin-to-creatinine ratio; ACEI, angiotensin-converting enzyme inhibitor; ARB, angiotensin II receptor blocker; BMI, body mass index; CAD, coronary artery disease; eGFR, estimated glomerular filtration rate; HbA1c, glycated hemoglobin; SGLT2, sodium-glucose cotransporter-2.

The unadjusted data show that ≥30% eGFR decline was uncommon in the normal-ACR group but substantially more frequent among participants with macroalbuminuria, particularly those receiving neither therapy. Because the treatment groups differed considerably in baseline ACR and other characteristics, these unadjusted comparisons should not be interpreted as treatment effects. The weighted and adjusted analyses therefore provide the principal inferential results **(Table S2).**

Supplementary Table S2. Unadjusted eGFR outcomes according to treatment group and baseline ACR category

Baseline ACR category	Treatment	N	Change in eGFR, median [IQR]	≥10% decline, n (%)	≥30% decline, n (%)
**Normal**	Neither	1,847	0.0 [−5.8, 4.2]	234 (13%)	20 (1%)
	ACEI/ARB only	1,625	0.0 [−7.2, 0.0]	341 (21%)	40 (2%)
	SGLT2 only	1,717	0.0 [−6.3, 0.0]	231 (13%)	26 (2%)
	Both	2,127	0.0 [−8.5, 0.0]	501 (24%)	91 (4%)
**Micro**	Neither	241	0.0 [−10.5, 0.0]	68 (28%)	22 (9%)
	ACEI/ARB only	573	−3.8 [−10.6, 0.0]	180 (31%)	41 (7%)
	SGLT2 only	237	0.0 [−8.7, 0.0]	61 (26%)	12 (5%)
	Both	1,249	−4.5 [−10.9, 0.0]	393 (31%)	83 (7%)
**Macro**	Neither	54	−10.0 [−22.8, 0.0]	32 (59%)	29 (54%)
	ACEI/ARB only	134	−9.0 [−20.5, 0.0]	80 (60%)	44 (33%)
	SGLT2 only	34	−4.5 [−12.1, 0.0]	13 (38%)	6 (18%)
	Both	414	−9.6 [−21.1, 0.0]	228 (55%)	103 (25%)

ACR, albumin-to-creatinine ratio; ACEI, angiotensin-converting enzyme inhibitor; ARB, angiotensin II receptor blocker; eGFR, estimated glomerular filtration rate; IQR, interquartile range; SGLT2, sodium-glucose cotransporter-2.

Supplementary Table S3. ACR outcomes according to treatment group and baseline ACR category

Baseline ACR	Treatment	N	Change in ACR, median [IQR]	≥50% ACR decline, n (%)
**Normal (<30 mg/g)**	Neither	1,842	0.4 [−1.1, 4.1]	**122 (6.6%)**
	ACEI/ARB only	1,624	1.2 [−1.7, 7.0]	**122 (7.5%)**
	SGLT2 only	1,716	1.4 [−0.8, 5.3]	**92 (5.4%)**
	Both	2,122	2.1 [−1.2, 9.7]	**147 (6.9%)**
**Micro (30–300 mg/g)**	Neither	240	−20.8 [−44.8, 0.0]	**110 (45.8%)**
	ACEI/ARB only	569	−13.6 [−45.4, 21.3]	**204 (35.9%)**
	SGLT2 only	235	−15.5 [−42.1, 5.7]	**100 (42.6%)**
	Both	1,242	−14.6 [−47.0, 28.9]	**450 (36.2%)**
**Macro (>300 mg/g)**	Neither	54	−300 [−795, 211]	**24 (44.4%)**
	ACEI/ARB only	134	−282 [−521, 0]	**59 (44.0%)**
	SGLT2 only	33	−236 [−401, −127]	**17 (51.5%)**
	Both	414	−263 [−646, 4]	**201 (48.6%)**

ACR, albumin-to-creatinine ratio; ACEI, angiotensin-converting enzyme inhibitor; ARB, angiotensin II receptor blocker; IQR, interquartile range; SGLT2, sodium-glucose cotransporter-2.

None of the pairwise treatment comparisons demonstrated a statistically significant difference after multiplicity adjustment. The comparison between SGLT2-only and combination therapy approached but did not meet conventional statistical significance **(Table S4).**

Supplementary Table S4. Pairwise comparisons between treatment groups for the primary outcome

Comparison	OR	95% CI	Adjusted P value
**Neither vs ACEI/ARB only**	0.95	(0.57–1.59)	0.994
**Neither vs SGLT2 only**	1.32	(0.64–2.72)	0.751
**Neither vs Both**	0.74	(0.48–1.12)	0.247
**ACEI/ARB only vs SGLT2 only**	1.39	(0.73–2.65)	0.546
**ACEI/ARB only vs Both**	0.78	(0.54–1.12)	0.285
**SGLT2 only vs Both**	0.56	(0.31–1.02)	0.061

Firth logistic regression was used as a sensitivity analysis because of sparse events in some treatment-by-ACR strata. The direction and magnitude of associations varied across ACR categories, although several estimates had wide confidence intervals. These analyses should be interpreted as sensitivity analyses rather than replacements for the primary weighted model **(Table S5).**

Supplementary Table S5. Firth logistic regression sensitivity analysis by baseline ACR category

Baseline ACR	Treatment	OR	95% CI	P value
**Normal**	ACEI/ARB only	1.54	0.90–2.69	0.114
	SGLT2 only	1.72	0.95–3.15	0.073
	Both	3.10	1.90–5.26	<0.001
**Micro**	ACEI/ARB only	0.65	0.37–1.16	0.140
	SGLT2 only	0.54	0.25–1.12	0.100
	Both	0.61	0.37–1.04	0.069
**Macro**	ACEI/ARB only	0.55	0.27–1.13	0.102
	SGLT2 only	0.37	0.12–1.03	0.058
	Both	0.47	0.24–0.90	0.022

ACR, albumin-to-creatinine ratio; ACEI, angiotensin-converting enzyme inhibitor; ARB, angiotensin II receptor blocker; CI, confidence interval; OR, odds ratio; SGLT2, sodium-glucose cotransporter-2.

After weighting, the descriptive outcome distributions remained variable across treatment groups. These estimates are presented to describe the weighted study population and should not be interpreted independently of the adjusted regression analyses **(Table S6).**

Supplementary Table S6. Weighted descriptive renal outcomes according to treatment group

Outcome	Neither	ACEI/ARB only	SGLT2 only	Both
**Mean change in eGFR ± SE**	−2.62 ± 0.43	−3.90 ± 0.33	−2.20 ± 0.39	−4.51 ± 0.21
**≥30% eGFR decline, %**	5.9	5.22	4.16	5.86
**≥10% eGFR decline, %**	19.32	25.22	18.74	26.89
**≥50% ACR decline, %**	**15.97**	**16.22**	**16.4**	**15.26**

In the unweighted Firth sensitivity analysis, combination therapy was associated with higher odds of ≥30% eGFR decline compared with neither therapy, whereas the ACEI/ARB-only and SGLT2-only groups did not show statistically significant associations. Because this sensitivity analysis does not incorporate the entropy-balancing weights used in the primary analysis, it should not supersede the primary adjusted estimates **(Table S7).**

Supplementary Table S7. Overall Firth logistic regression sensitivity analysis

Treatment	OR	95% CI	P value
**ACEI/ARB only**	1.18	0.85–1.63	0.327
**SGLT2 only**	0.83	0.55–1.24	0.372
**Both**	1.52	1.14–2.06	0.004

There was evidence of effect modification by baseline ACR category (global interaction P=0.029). This indicates that the association between treatment group and ≥30% eGFR decline differed according to baseline ACR category. The individual interaction terms should not be interpreted as the treatment effects within each ACR stratum; those stratum-specific estimates should instead be obtained from appropriately parameterized models **(Table S8)**.

Supplementary Table S8. Treatment-by-baseline ACR interaction for ≥30% eGFR decline

Interaction term	OR	95% CI	P value
**ACEI/ARB × Microalbuminuria**	0.351	0.137–0.903	0.030
**SGLT2 × Microalbuminuria**	0.389	0.132–1.140	0.086
**Both × Microalbuminuria**	0.209	0.095–0.459	<0.001
**ACEI/ARB × Macroalbuminuria**	0.407	0.139–1.190	0.100
**SGLT2 × Macroalbuminuria**	0.178	0.040–0.796	0.024
**Both × Macroalbuminuria**	0.202	0.079–0.517	<0.001
**Global treatment × ACR interaction**	—	—	**0.029**

Entropy-balancing weights produced effective sample sizes below the nominal sample sizes, particularly for the SGLT2-only group. The maximum weight of 137 in the SGLT2-only group indicates potential weight instability and should be considered when interpreting the weighted estimates. Formal standardized mean differences before and after weighting should accompany this table to demonstrate covariate balance **(Table S9)**.

Supplementary Table S9. Entropy-balancing weight diagnostics

Treatment group	N	Mean weight	SD	Median	Min	Q1	Q3	Max	Effective sample size
**Neither**	2,142	4.79	2.56	4.18	0.957	3.35	5.39	34.0	1,665
**ACEI/ARB only**	2,332	4.40	2.49	3.83	1.15	2.93	5.07	26.1	1,766
**SGLT2 only**	1,988	5.16	4.90	4.56	0.797	3.38	5.96	137.0	1,045
**Both**	3,790	2.70	1.03	2.62	0.025	1.98	3.34	8.12	3,307
